# Case report and literature review: Novel compound heterozygous *FIG4* variants causing both of peripheral and central nervous system defects

**DOI:** 10.3389/fped.2022.1008251

**Published:** 2022-10-21

**Authors:** Yonglin Yu, Hongwei Yin, Changli Ma, Xiaoyi Jia, Wencong Chen, Haifeng Li, Ke Wu

**Affiliations:** ^1^ Department of Rehabilitation, The Children’s Hospital Zhejiang University School of Medicine, National Clinical Research Center for Child Health, Hangzhou, China; ^2^ Chigene (Beijing) Translational Medical Research Center Co Ltd, Beijing, China; ^3^ Prenatal Diagnosis Center, Yiwu Maternity and Child Health Care Hospital, Yiwu, China

**Keywords:** *FIG4* gene, Charcot-Marie-Tooth disease, central nervous system, cognitive deficits, compound heterozygous variants

## Abstract

**Background:**

Pathogenic variants in the *FIG4* gene have been described to be associated with a diverse spectrum of syndromes, such as autosomal recessive bilateral temporooccipital polymicrogyria (OMIM 612691), autosomal dominant amyotrophic lateral sclerosis-11 (ALS11; OMIM 612577), autosomal recessive Charcot-Marie-Tooth disease, type 4J (CMT4J; OMIM 611228), and autosomal recessive Yunis-Varon syndrome (YVS; OMIM 216340). Heterozygous *FIG4* variants are responsible for ALS11 characterized by progressive muscular weakness, atrophy, and bulbar palsy. CMT4J is a disorder of peripheral nervous system defects mainly presenting with a highly variable onset of proximal and/or distal muscle weakness. YVS is a disorder of severe neurological involvement with central nervous system (CNS) dysfunction and extensive skeletal anomalies.

**Case Presentation:**

We reported two Chinese siblings born with a weakness in all limbs. They experienced rapidly progressive weakness in distal limbs. At the age of 6 years, the elder brother presented with severe scoliosis and cervical kyphosis. They both had global developmental delay and a CNS involvement with cognitive deficits and swallowing problems. Genetic screening in the patients' family for inherited diseases was recommended. Novel compound heterozygous variants in the *FIG4* gene (c.2148delTinsAA and c.317A > G) were found by whole-exome sequencing in the patients. These variants were confirmed by Sanger sequencing in family members.

**Conclusions:**

Herein, we reported two Chinese male patients with CMT4J who presented with abnormal CNS features. CMT4J with CNS involvement has been very rarely reported. We hoped this study could expand the phenotypic and genetic spectrum of FIG4-related diseases. And we helped physicians to understand the genotype–phenotype correlation.

## Introduction

Patients with Charcot-Marie-Tooth disease, type 4J (CMT4J) predominantly presented with progressive weakness of distal and/or proximal muscle, significant motor dysfunction, and variably progressive sensory loss. Nerve biopsies indicated demyelination and axonal loss (1). CMT4J accounted for 0.3% of 17,880 individuals with neuropathy (2). The age of onset varied from early childhood to the sixth decade. The congenital or infantile onset of CMT4J has been rarely reported. Yunis-Varon syndrome (YVS) was a rare and severe heterogeneous autosomal recessive disorder characterized by severe skeletal abnormalities, brain malformations, retinopathy, and facial dysmorphisms. YVS also presented with central nervous system (CNS) anomalies like global developmental delay, and feeding and swallowing difficulties. Less than 30 cases of YVS have been reported (3). YVS usually led to lethality in infancy or early childhood. While most CMT4J patients did not exhibit CNS deficits, recent studies reported that rare CMT4J cases involved abnormal CNS. Zimmermann et al. (4) described five patients with a phenotypical continuum between CMT4J and YVS. *FIG4* gene encoded the protein polyphosphoinositide phosphatase, which catalyzed the dephosphorylation of phosphatidylinositol 3,5-bisphosphate [PtdIns(3,5)P2] to form phosphatidylinositol 3-phosphate (PtdIns3P) (5). *Fig4*^−/−^ null mice studies showed defective repair of CNS myelin and damage of myelinated axons in the mature peripheral nervous system (PNS) (6). Homozygous or compound heterozygous deleterious *FIG4* variants causing a phenotypical of combined PNS and CNS defects have been very rarely reported. Herein, we report two Chinese siblings with compound heterozygous deleterious *FIG4* variants presented with abnormal PNS and CNS features.

## Case presentation

**Figure 1:**
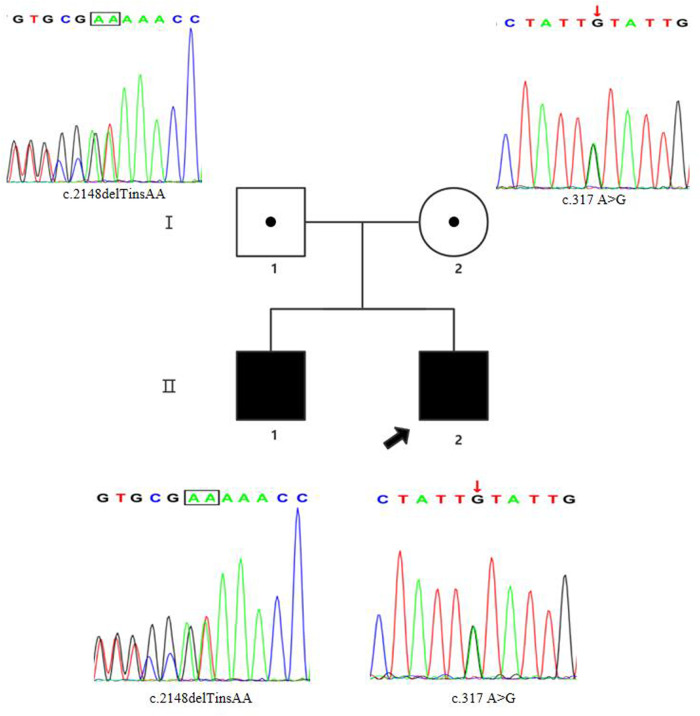
Pedigree chart and Sanger sequencing results of the family members. The proband was the younger brother (marked with the black arrow). The two siblings carried the compound heterozygous *FIG4* variants. The results of Sanger sequencing indicated that the father was a heterozygous carrier of the c.2148delTinsAA (p.P718Tfs*3) variant (marked with the black box), and the mother was a heterozygous carrier of the c.317A > G (p.Y106C) variant (marked with the red arrow).

The pedigree had two male patients (Figure 1), who were the children of phenotypically normal Chinese parents. The parents did not have consanguineous relation and their family history was not notable. No neurologic disease was reported in the parent's family. The two brothers were naturally conceived with uneventful normal pregnancy. The elder brother (**II1**) was not treated and did not receive rehabilitation therapy in a local hospital since he was born with a weakness in all limbs, so we could not get his detailed clinical data. At the age of 6 years, the proband (**II2**) was born at 40 weeks gestation.

**Figure 2:**
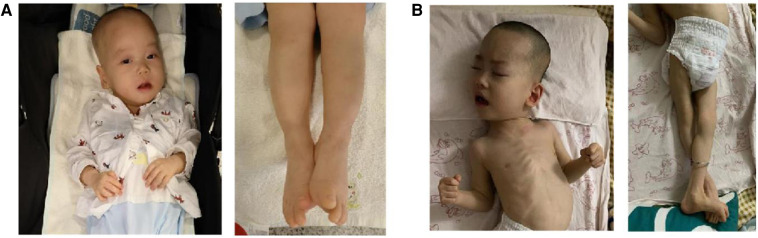
Upper and lower limbs of the two siblings. (**A**) The proband (1 year old); (**B**) the elder brother (7 years old).

Since the proband was born, he was hospitalized for weakness of all extremities and received treatment for 7 days in the local neonatology department. At the age of 3 months, he was treated in a local rehabilitation institution for “developmental retardation”, and there was no significant improvement. At the age of 10 months, the boy was referred to the rehabilitation department of our hospital. He had an occipital frontal circumference (OFC) of 44.5 cm. No seizures occurred. Scattered red macules could be seen on the skin of the whole body. There were no distinctive facial features except for a high-arched palate and dental dysplasia. He had problems with swallowing and dental growth. Now, he could take small amounts of solids and liquids. No congenital malformations of the scrotum and cryptorchidism were found in both patients. His eyes could track objects, and he responded to sound or simple verbal commands. He could not lift his head, roll over, stand on four limbs, or sit alone without support. He was not able to make a fist or grasp objects. His hands showed thumb adduction deformity. For now, he still suffered from constipation. As their mother recalled, the elder brother had similar symptoms and disease processes. Now he could not walk alone (Figure 2). A nerve biopsy was offered but refused by the parent.

The modified Ashworth scale (MAS) (7) is clinically used for measuring the increase of muscle tone. The MAS of both upper and lower limbs was 1. A neurological examination revealed decreased muscle strength (Medical Research Council Scale for Muscle Strength was Grade 4/5 in all extremities). Bilateral knee tendon reflexes were positive. The Babinski sign, the Moro reflex, and the asymmetrical tonic neck reflex (ATNR) were all present. The ankle clonus reflex was absent.

Blood laboratory tests revealed a low hemoglobin count of 80 g/L (120–165 g/L), low mean corpuscular volume (MCV) of 48.2 fL (82–100 fL), low mean corpuscular hemoglobin (MCH) of 13.6 pg (26–32 pg), low mean corpuscular hemoglobin concentration (MCHC) of 282 g/L (316–354 g/L), a low IgG level of 1.60 g/L (7–16 g/L), and an elevated IgE level of 1020.0 IU/mL (20–200 IU/mL). Allergy testing showed that he was allergic to the dander of dogs. Thyroid function testing revealed that an elevated triiodothyronine (T3) level of 2.39 nmol/L (0.98–2.33 nmol/L) and an increased thyroxine level of 180.03 nmol/L (62.68–150.80 nmol/L).

**Figure 3:**
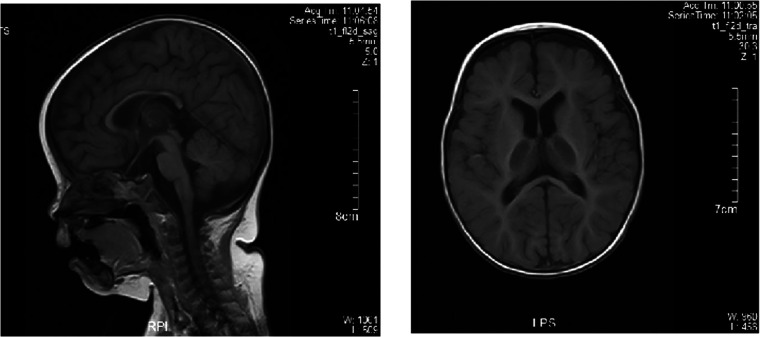
Brain MRI of the proband. Brain MRI showed the normal bilateral lateral ventricle and slightly thinner corpus callosum.

Echocardiography indicated the mildly regurgitant tricuspid valve. A nerve conduction study of all four limbs revealed the slightly decreased motor nerve conduction velocity (MCV) in the left tibial and bilateral median nerves and the prolonged F-wave latencies of the left tibial nerve. Brain magnetic resonance imaging (MRI) revealed a slightly thinner corpus callosum, and symmetrical long T1 and T2 signal changes in the ventral medulla oblongata and bilateral thalamus (Figure 3). Brain MRI of the elder brother was not available.

**Figure 4:**
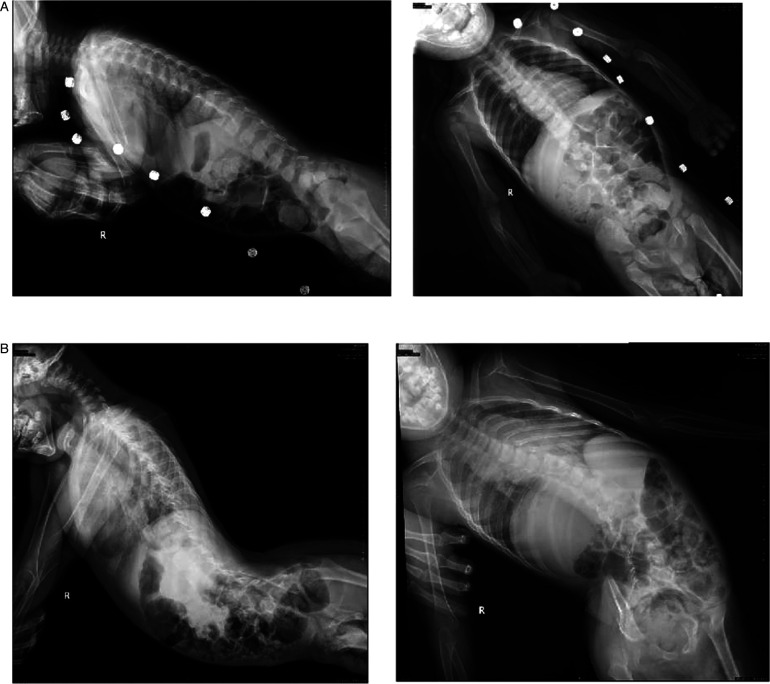
The spine x-rays. (**A**) The spine x-rays of the proband; (**B**) the spine x-rays of the elder brother. The spine x-rays showed that the spine of the proband was normal and the elder brother had severe scoliosis and cervical kyphosis.

The spine x-rays of the two siblings showed that the spine of the proband was normal (Figure 4A) and the elder brother had severe scoliosis and cervical kyphosis (Figure 4B). The hand x-rays of the two siblings did not show abnormalities.

The Grifths mental development scales for China (GDS-C) (8) are used to assess the development of children from birth to 8 years across six separate subscales: locomotor (A), personal-social (B), language (C), eye-hand coordination (D), performance (E), and practical reasoning (F). Developmental curves with respect to all six subscales together with the general quotient (GQ) were plotted. The proband had a GQ of 12.70 (The raw scores of the six subscales were all lower than the 1st percentile). The low score of GQ suggested that he was significantly delayed in his cognitive and physical development. The GQ of the elder brother was not available, because of his noncooperation. For this reason, genetic screening in the patient's family for inherited diseases was recommended.

## Whole-exome sequencing (WES)

The parents signed informed consent for genetic analysis. Our legal ethics committee approved this genetic study. Genomic DNA was extracted from the peripheral blood of the patients and phenotypically normal parent for WES. The xGen™ Exome Research Panel v2 (designed by Integrated DNA Technologies) was used for WES. Quality control (QC) of the DNA library was performed using an Agilent 2100 Bioanalyzer System. DNA nanoball (DNB) preps of clinical samples were sequenced on ultra-high throughput DNBSEQ-T7 platform (MGI, Shenzhen, China) with paired-end 150 nt strategy following manufacturer’s protocol.

## Bioinformatic analysis

Sequencing data (a total of 19,136 genes) was analyzed according to our in-house (Chigene Translational Medicine Research Center) procedures. Adapters and low-quality reads were removed, and then data quantity and data quality were statistics. The trimmed reads were then mapped to UCSC GRCh37/hg19 reference genome using the software Burrows-Wheeler Aligner (BWA). GATK software was used for single nucleotide polymorphisms (SNP) and short (<50 bp) insertion/deletion (indel) calling. Samtools and Picard software packages were used to generate clean Bam data by removing duplicate data. Variants were annotated for analysis using the single nucleotide polymorphism database (dbSNP), gnomAD exomes database, and Chigene in-house minor allele frequency (MAF) database. Tools of pathogenicity prediction like SIFT, PolyPhen-2, MutPred, MutPred, and MutationTaster were used for predicting the possible impact of variants. Splice site variants were investigated with prediction programs like SpliceSiteFinder, MaxEntScan, Human Splice Finder, and SpliceAI. As a prioritized pathogenicity annotation to the American College of Medical Genetics and Genomics (ACMG) guidelines, Online Mendelian Inheritance in Man (OMIM), Human Gene Mutation Database (HGMD), and ClinVar databases were used as conferences on pathogenicity of every variant.

## Variant classification

As per the guidelines of ACMG for interpreting sequence variants, variants were classified. Classification considered MAF and pathogenicity prediction of variants, disease mechanism, clinical phenotypes, literature evidence, and evolutionary conservation. Variants with MAF > 1% in population databases or predicted benign/neutral pathogenicity were excluded from further investigation. A further investigation focused on the genotype–phenotype correlations, evolutionary conservation of mutant sites, literature evidence, and disease mechanism.

## Genetic analysis

Sanger sequencing (BigDye Terminator v3.1 Cycle Sequencing Kit and ABI 3730 Applied Biosystem) was used for further verification. We finally identified novel compound heterozygous *FIG4* variants (c.2148delTinsAA and c.317A > G) (NM_014845.6). Cosegregation analysis was performed among family members (other available affected/unaffected relatives). The results of Sanger sequencing indicated that the father was a heterozygous carrier of the c.2148delTinsAA (p.P718Tfs*3) variant, and the mother was a heterozygous carrier of the c.317A > G (p.Y106C) variant (Figure 1; Table 1).

**Table 1:** Genomic findings and variants interpretation.

*FIG4* variants (NM_014845.6)	Genomic location (hg19)	Zygosity	Parent of origin	Interpretation
c.317 A > G (p.Y106C)	chr6:110048339	Het	Maternal	Likely pathogenic (PM1, PM2, PP3, PP4)
c.2148delTinsAA (p.P718Tfs*3)	chr6:110110848	Het	Paternal	Likely pathogenic (PVS1, PM2, PP4)

Criteria: PVS1, null variant; PM1, variants located in a mutational hot spot and/or critical and well-established functional domain without benign variation; PM2, variants were absent from controls; PP3, multiple lines of computational evidence supported a deleterious effect on variants; PP4, patient’s phenotype was highly specific for a disease with a single genetic etiology.

The allele frequency of heterozygous c.317A > G was 1/18,392 in the East Asian population of the gnomAD exome database (PM2). The position of this variant was strongly conserved (PhyloP100way score of c.317A > G was 8.99 and greater than 7.2. PhyloP100way scores are based on multiple alignments of 99 vertebrate genome sequences to the human genome). The c.317A > G (p.Y106C) variant was located in a critical and well-established functional domain (SAC phosphatase domain of FIG4 protein) (PM1). *In silico* predictive algorithms of pathogenicity (SIFT, PolyPhen-2, MutPred, MutPred, and MutationTaster) showed that the missense variant was damaging (PP3). A homozygous c.317A > G variant was identified in three CMT4J siblings who presented with spastic quadriplegia, epilepsy, and global developmental delay (9). Chaudhuri et al. interpreted this variant as a likely pathogenic variant (PS4; PM2; PP1; PP3; PP4) (9).

**Figure 5:**
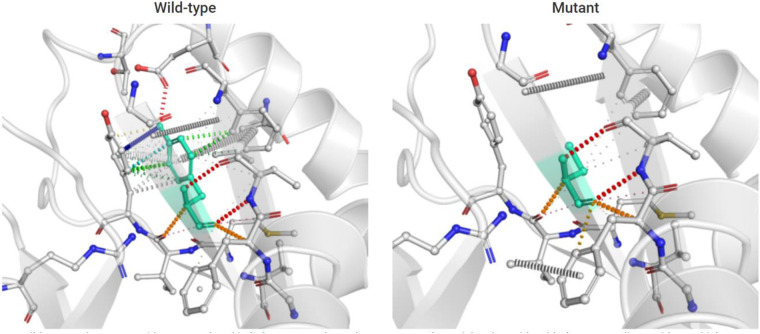
Prediction of interactomic interactions of the wild-type and mutant site (Y106 residue mapped to Y94 in the PDB structure). Residues in the wild-type and mutant sites were colored in light green and shown as sticks. The respective chemical interactions were labeled as dotted lines and colored as follows: hydrogen bonds—(red), weak hydrogen bonds—(orange), hydrophobic contacts—(green), amide-amide contacts—(blue), and ionic interactions—(gold). Amino acid residues were also colored according to type, namely nitrogen (blue), oxygen (red), and sulfur (yellow). In comparison to the wild-type site, some interactions (hydrophobic contacts, amide-amide contact, and ionic interactions) were observed to be lost in the mutant site.

DynaMut (a web server) (10) is a well-established normal mode approach. We used it to visualize and assess the stability and interactomic interactions of the mutant protein. We put information into DynaMut as follows: wild-type structure (PDB accession code:7K1W), variant detail (Y94C, chain F) (Y106 residue mapped to Y94 in the PDB structure). The prediction outcome of stability was ΔΔ*G*: −0.304 kcal/mol (destabilizing). The Y94 residue in the mutant site was observed to form residue interactions (colored in light green) with its surrounding residues, whereas some interactions (hydrophobic contacts, amide-amide contacts, and ionic interactions) were observed to be lost in the mutant site (Figure 5).

The c.2148delTinsAA variant had not been reported in disease databases (ClinVar, HGMD, OMIM) or registered in population databases (1000 Genomes Project, gnomAD, dbSNP) (PM2). It was a null variant (frameshift mutation) in the *FIG4* gene where loss-of-function (LOF) is a known mechanism of CMT4J (PVS1). As per the guidelines of ACMG for interpreting sequence variants, the compound heterozygous variants were likely pathogenic.

Conventional G-banded chromosome analysis of the patients showed a 46, XY karyotype. The results of the genetic metabolic disease screening of the patients were all normal.

## Literature review

We searched PubMed, HGMD, and OMIM databases using “Charcot-Marie-Tooth disease, type 4J”, “Yunis-Varon syndrome”, and “*FIG4*” as keywords. The search time was from the establishment of the databases to 30 June 2022. Patients with *FIG4* variants presenting with a phenotypical continuum between CMT4J and CNS involvement were included in this review. Ten documents were retrieved. Thirty CMT4J cases with homozygous or compound heterozygous *FIG4* variants that were involved with CNS anomalies have been reported (Table 2). The p.I41T variant was a recurrent mutation reported in a number of unrelated families (11). It has been estimated that the population frequency of this allele was 0.001 (1).

**Table 2:** CMT4J cases with CNS involvement.

Reference	Ethnicity	Number of reported cases	Age at onset	Age at genetic diagnosis	*FIG4* variants (NM_014845.6)	Brain MRI	CNS involvement
Orengo et al. (12)	ND	1	12 years	52 years	c.122T > C (p.I41T), c.1949-10T > G	Diffuse cortical atrophy with pronounced focal left temporal lobe atrophy	Aphemia, mild cognitive impairment, slow resting hand tremor
Posada et al. (13)	Caucasian	1	14 years	51 years	c.122T > C (p.I41T), c.1447C > T (p.R483*)	Normal	Parkinsonism
Nicholson et al. (1)	Australian	2	ND	46 years, 54 years	c.122T > C (p.I41T), c.904G > A (p.E302K); c.122T > C (p.I41T), c.759delG (p.F254Sfs*8)	Atrophy of frontal and parietal lobes and cerebellum	Parkinsonism
Chaudhuri et al. (9)	South Asian	3	Birth	4 years, 7 years, 10 years	Homozygous c.317A > G (p.Y106C)	Bilateral thalamic hyperintensities	DD, seizures, spastic quadriplegia
Hu et al. (14)	ND	3	Birth, 12 years	8 years, 11 years, 52 years	c.122T > C (p.I41T), c.1949-10T > G; c.122T > C (p.I41T), c.1373dup (p.L458Ffs*5); c.2459 + 1G > A,c.831_838del (K278Wfs*6)	White matter changes	Parkinson, ID
Baulac et al. (15)	Moroccan	3	ND	23 years, 35 years, 55 years	Homozygous c.2348A > T (p.D783V)	Bilateral occipital polymicrogyria, cortical abnormality, enlarged cerebral ventricles	Seizures, psychiatric manifestations
Wright et al. (16)	Pakistani South Asian	4	2 m, 7 m, 18 m	3 years, 11 years, 12 years	Homozygous c.506A > C (p.Y169S)	Cerebellar atrophy, widespread white matter abnormalities, atrophy typical of HOD	DD, autism, severe ID, microcephaly, feeding and swallowing difficulties
Zimmermann et al. (4)	ND	5	5 years, 40 years, 41 years, 65 years	20 years, 30 years, 52 years, 56 years, 67 years	c.122T > C (p.I41T), c.2459 + 1G > A; c.122T > C (p.I41T), c.2188dup (p.S730Kfs*3); c.122T > C (p.I41T), c.1141C > T (p.R381*); homozygous deletion of exon 21	Global brain atrophy, diffuse hyperintensities in brain stem, leukoencephalopathy	DD, parkinsonism, cerebellar syndrome, seizures, hypacusis, retinal and optic atrophy
Lenk et al. (17)	ND	7	5 m, 9 m, 1 year	3 years, 4 years, 6 years, 8 years, 11 years	c.2459 + 1G > A, c.737G > C (p.W246S); c.1475G > C (p.R492P), c.2439_2441del (p.E813del); homozygous c.506A > C (p.Y169S); homozygous c.2459 + 1G > A	Diffuse hypomyelination, abnormal signal in the internal capsule and cerebral white matter, reduction in white matter bulk, mild ventricular dilatation	DD, feeding difficulties, cognitive impairment, autistic features, maculopathy, optic and retinal atrophy
Michaelidou et al. (18)	ND	1	50 years	63 years	c.122T > C (p.I41T); c.1795delC (p.H599fs*24);	Normal	Parkinsonism
Our patients	Chinese	2	Birth	10 m, 7 years	c.317A > G (p.Y106C), c.2148delTinsAA (p.P718Tfs*3)	Slightly thinner corpus callosum	DD, severe cognitive impairment, feeding and swallowing difficulties

M, male; F, female; y, years; m, month; ND, not done or no data; DD, developmental delay; HOD, hypertrophic olivary degeneration; ID, intellectual disability.

## Discussion

**Figure 6:**
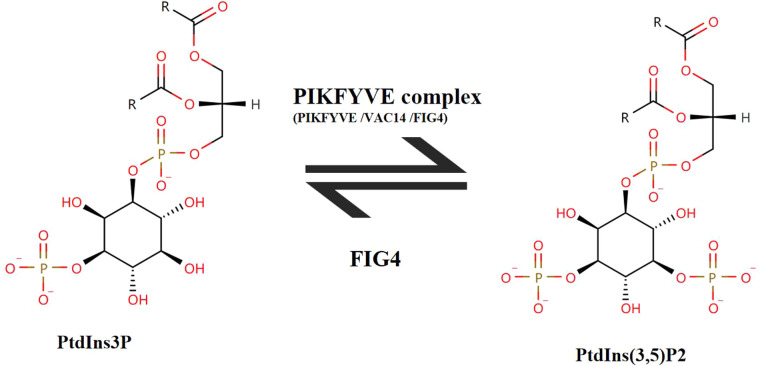
The transition between PtdIns(3,5)P2 and PtdIns3P. FIG4 catalyzes the dephosphorylation of PtdIns(3,5)P2 to form PtdIns3P. PIKFYVE complex consists of three subunits (PIKFYVE, VAC14, FIG4). PIKFYVE is the sole enzyme that catalyzes the phosphorylation of PtdIns3P on the fifth hydroxyl of the myo-inositol ring to form PtdIns(3,5)P2.

The human *FIG4 phosphoinositide 5-phosphatase* (*FIG4*) gene is located in chromosome 6q21. The transcript of *FIG4* (NM_014845.6) has 23 exons, transcript length of 3,025 base pairs, and translation length of 907 amino acids. The polyphosphoinositide phosphatase protein (FIG4) [UniProtKB-Q92562] belongs to the SAC domain-containing protein family. Cytoplasmic expression of FIG4 is in most of the tissues. The Human Protein Atlas (HPA) shows that FIG4 is highly expressed in the brain, endocrine tissues, skin, and respiratory system. FIG4 catalyzes the dephosphorylation of PtdIns(3,5)P2 to form PtdIns3P. PtdIns3P is a phospholipid in cell membranes that helps to recruit a range of proteins. PIKfyve complex as a lipid kinase is conserved in all eukaryotes and phosphorylates PtdIns3P to PtdIns(3,5)P2 (19) (Figure 6). It is the only source of PtdIns(3,5)P2 (5). PtdIns(3,5)P2 is a low-abundance signaling lipid that maintains endomembrane homeostasis (20).

FIG4 contains an N-terminal SAC phosphatase domain of 331 amino acids (residues 93–423). Most disease-causing *FIG4* variants occurred within the SAC phosphatase domain. Axonal degeneration of motor neurons and demyelination of Schwann cells were observed in *Fig4*-null mice (21). Neonatal *Fig4*^−/−^ mice exhibited hypomyelination, spongiform degeneration of neural tissues, intention tremor, and juvenile lethality (6). Neurons are particularly sensitive to reduced abundance of PtdIns(3,5)P2 that is localized to the cytoplasmic surface of endolysosomal vesicles. The kinase PIKFYVE complex regulates the abundance of PtdIns(3,5)P2. The other two components of the PIKFYVE complex are the PIKFYVE activator VAC14 and the phosphoinositide 5-phosphatase FIG4 (Figure 6). The phosphatase FIG4 is bound to the scaffold protein VAC14. A 50% reduction in the level of PtdIns(3,5)P2 was observed in *Fig4*^−/−^ fibroblasts (22). Thus, *FIG4* variants may lead to reduce the activity of FIG4 protein, and then LOF of FIG4 protein damages the PIKFYVE complex. *FIG4* variants were associated with dominant ALS11. Dorrity et al. (23) identified the conserved position of the “dominant negative fragment” in yeast. Chow et al. suggested that heterozygous missense variants of the *FIG4* gene could exert their effects by a dominant negative mechanism *via* competition with the wild-type protein for incorporation into the multimeric PtdIns(3,5)P2-regulatory complex (24). But in literature review of ALS11 cases, the variants (Q403X and R183X) were identified in both familial ALS11 cases and CMT4J cases (25). We also did not find “dominant negative fragment” or specific/hot mutation regions/domains for ALS11, CMT4J, and YVS-related *FIG4* variants in literature review of Liu et al. (26) and Umair et al. (27). Thus, LOF is a known mechanism of *FIG4* gene-related recessive diseases.

As we could see from Table 2, 58% (18/31) of cases were affected with CTM4J and CNS abnormalities during the infant period. The age of onset of CTM4J varied from early childhood to the sixth decade. The congenital or infantile onset of CMT4J has been rarely reported. YVS was caused by null variants in *FIG4*, which resulted in complete LOF (28). Compared with CTM4J, YVS was a more severe disorder characterized by CNS problems and skeletal abnormalities (cleidocranial dysplasia, digital anomalies). Enlarged cytoplasmic vacuoles were found in neurons, muscles, and cartilage. YVS patients most frequently died during the neonatal period or in early childhood (27). Our patients had global developmental delays, but they were unlike classical YVS. They did not have cleidocranial dysplasia, absent thumbs, dysmorphic features, aphalangia of fingers and toes, or hearing loss, but they presented with partial symptoms of YVS like severe developmental delay, intellectual disability, and feeding and swallowing difficulties. Besides, the symptoms (parkinsonism, autistic features, maculopathy, optic and retinal atrophy, microcephaly, aphemia, spastic quadriplegia, and psychiatric manifestations) have been reported in CMT4J patients. Thus, it is should be noted for clinicians to recognize the phenotypic variability of CMT4J. In the adult PNS, FIG4 was required to protect myelinated axons from Wallerian degeneration; in the CNS, FIG4 was required for myelin repair but not maintenance. The greater vulnerability of the PNS to FIG4 deficiency in the mouse was consistent with clinical observations in patients with CMT4J (29). Thus, CMT4J patients with CNS defects have been rarely reported.

Reducing the intralysosomal Ca^2+^ by application of TRPML1 synthetic ligand rescued abnormal lysosomal storage in *Fig4*^−/−^ culture cells. It might be a potential therapy against diseases with FIG4 deficiency (30). Now therapies for CMT4J like gene silencing, gene replacement therapies, and small molecule treatments are in preclinical testing, some therapies reached the clinical trial stage (31).

In conclusion, we described that two Chinese siblings with compound heterozygous deleterious *FIG4* variants presented with abnormal PNS and CNS features. Then we reviewed related literature and helped clinicians to be aware of the wide phenotypic spectrum of *FIG4*-related diseases. We hoped that this study could be helpful to early diagnosis and treatment.

## Data Availability

The datasets presented in this study can be found in online repositories. The names of the repository/repositories and accession number(s) can be found in the article/Supplementary Materials.
